# Application of PLLA (Poly-L-Lactic acid) for rejuvenation and reproduction of facial cutaneous tissue in aesthetics: A review

**DOI:** 10.1097/MD.0000000000037506

**Published:** 2024-03-15

**Authors:** Yin-Jie Ao, Yan Yi, Guo-Hui Wu

**Affiliations:** a Zhengxing Stomatological Hospital, Yichun City, Jiangxi Province, P.R. China; b Ophthalmology Hospital Affiliated to Nanchang University, Nanchang City, Jiangxi Province, P.R. China.

**Keywords:** application, Poly-L-lactin acid (PLLA), rejuvenation

## Abstract

Poly-L-lactin acid (PLLA) has been widely used in the field of bio-medicine. In 2004, as an injectable material, PLLA was approved by the FDA to treat AIDS-related facial atrophy. Since then, several injectable stuffs containing PLLA have been approved for marketing in various countries and regions. Recently, PLLA has often been used to treat facial rejuvenation problems like cutaneous depressions and static wrinkles which always induce unsatisfactory facial expression. This review introduces the physicochemical properties, regeneration stimulating mechanism, applications in aesthetics and injectable comorbidity of PLLA.

## 1. Introduction

In 1954, as an absorbable, semi-permanent, bio-compatible and immunologic inertia injectable filler, Poly-L-lactin acid (PLLA) was invented by French chemists.^[1,2]^ European countries applied PLLA earlier and it has achieved mature commercialization nowadays.^[3]^ Brands of PLLA fillers mainly include Sculptra (France), Derma Veil (America), AestheFill (South Korea), Rebron (China), EVOPLLA (China), etc.^[4–6]^ Seulptra is the most prestigious one among them.^[7]^ In 2004, Sculptra was approved by FDA to treat AIDS related facial fat atrophy.^[8]^ In 2009, it was approved to treat age-related wrinkles.^[9]^ In 2021, domestic PLLA filler was approved by Chinese National Medical Products Administration for marketing. In other fields of medicine, PLLA has also been used as material to produce absorbable sutures, orthopedic fixation devices, stents of urethral and tracheal, dental implants and vaccine carriers.^[10,11]^ In dermatology, PLLA can be applied in facial rejuvenation aspects such as facial volume filling, scarring plasty and wrinkles relieving through stimulates collagen regeneration.^[12,13]^ According to Vleggaar D, PLLA could induce collagen regeneration and be metabolized to carbon dioxide and water slowly.^[14]^ Recent studies have found that even if original PLLA be degraded completely, the regenerated collagen fibers can still function for a long time.^[15]^ The material has long-lasting effectiveness and a high level of safety and satisfaction.^[2]^

## 2. Physicochemical property of PLLA

**Figure 1.**
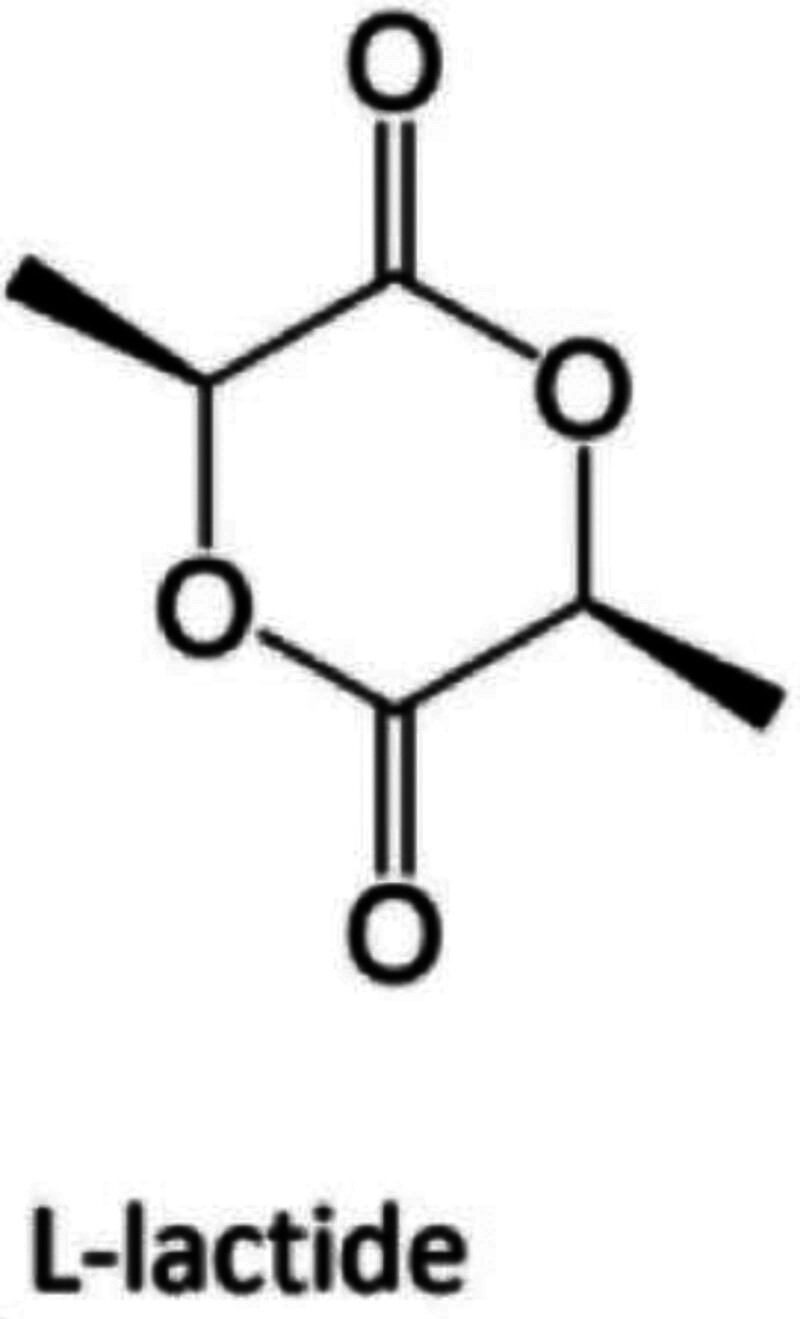
Chemical formula of poly-L-lactic.

**Figure 2.**
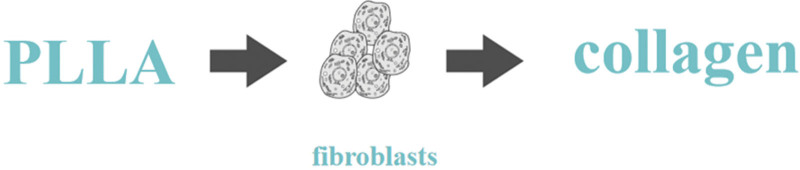
Function of poly-L-lactic *PLLA: poly-L-lactic.

Polylactic acid (PLA) is a poly-α-hydroxy acid synthesized from lactic acid (LA) which exists in 2 optically active stereoisomers, namely, L-LA and D-LA (S and R in absolute configuration, respectively).^[16]^ The polymerization of optically pure L- and D-lactide yields isotactic homopolymers of Poly-(L-lactide) (PLLA) and Poly-(D-Lactide), respectively.^[16]^ At present, PLLA is the main material of PLA series in the market of medical cosmetology.^[7]^ Injectable PLLA materials are generally lyophilized powder containing PLLA microparticles (Fig. 1).^[7]^ Taking Sculptra as an example, each vial of Sculptra incorporates PLLA microparticles, carboxymethylcellulose and nonpyrogenic mannitol, the rest components may benefit from stability of the microparticles as well as swelling mitigation after injection.^[7]^ As a member of alpha-hydroxy acids, PLLA is a kind of no-toxic and absorbable polymer with molecular weight from 40kDa to 50kDa.^[17]^ It needs to be resuspended with 3-5ml saline and be finished injection within 72 hours.^[17]^ Notably, the molecular weight of PLLA not only can prevent it be phagocytosed by dermal macrophages and capillaries, but can make it easier for clinicians to injected by 27G syringe.^[14,18]^ The immediate filling effect of PLLA will fade away after 1 week when solvent is absorbed.^[17]^ However, the remaining PLLA microparticles will be encapsulated by proliferated macrophages which be induced by subclinical inflammation of foreign body (Fig. 2).^[19]^ According to Gottfried et al, PLLA microparticles were still be encapsulated by lymphocytes and macrophages 3 months after injection.^[20]^ PLLA will be degraded into LA and then be metabolized to carbon dioxide and water, or be synthesized with glucose.^[19]^ Brady JM also figured out the final metabolites of PLLA are water and carbon dioxide, and the series of reactions are irreversible.^[19]^ Through rodents experiments, Brady JM verified PLLA can induce the proliferation of lymphocytes and macrophages, thereby stimulate the proliferation of fibroblasts.^[19]^ According to Vleggaar D, in the first 6 months after injection, with the foreign body inflammation decreases gradually, the type I collagen will proliferate gradually, which can maintain for 2 years or longer.^[14]^

## 3. PLLA stimulates proliferation and regeneration of cutaneous collagen

Unlike traditional fillers which rely on volume augmentation alone to achieve filling effect, PLLA stimulates proliferation of collagen by regenerating fibroblasts.^[21]^ Instant filling effect of PLLA will disappear from 3 to 7 days gradually after injection with the absorption of the solvent.^[22]^ PLLA microparticles will colonize in situ and begin to work.^[23]^ 1 month after injection, PLLA microparticles are encapsulated by macrophages and lymphocytes which are generated by foreign body inflammation.^[24]^ 3 months after injection, PLLA microparticles shrink gradually and be hydrolyzed into PLA or LA.^[25]^ After phagocytosis, the final metabolites are carbon dioxide and water.^[26]^ The reaction will stimulate the proliferation of fibroblasts.^[26]^ Six months after injection, the proliferation rate of fibroblasts reach climax and begin to secrete collagen vastly, mainly type I collagen, to fill the tissues.^[27]^ To brief the comprehensive reaction chain, the injected PLLA microparticles will activate subclinical foreign body inflammation, which will lead to collagen proliferation through fibroblasts colonization and regeneration.^[24]^ The ultimate metabolites are carbon dioxide and water.^[24]^ In vitro experiments have also confirmed the stimulating effect of PLLA concerning collagen synthesis.^[28,29]^ It is noteworthy that fibroblasts function as a regulator of collagen proliferation in these reactions, Which means when original PLLA microparticles are totally metabolized, the regenerated fibroblasts can still function precisely for at least 2 years or long.^[30]^ Whats more, unlike the growth factor materials, in which the tissues will proliferate in a rate of high multiplication for 10 years or even longer, which will result in catastrophic outcomes for most of patients.^[31]^ The collagen proliferation of PLLA will be subtly administered by fibroblasts and then provide a gentle and controllable result.^[32,33]^ In addition, according to Hanako et al, type III collagen will be found 16 weeks after injection.^[34]^ Type III collagen, for its production is limited in adult, which also be addressed as “baby collagen.”^[35]^ Simultaneously, for its distribution mainly between epidermis and dermis, which also be addressed as “microcollagen.”^[35]^ Type III collagen functions as subcutaneous scaffold, which relates to the formation of superficial facial static wrinkles.^[36]^ Therefore, PLLA can induce proliferation of type I/III collagen and benefit for refreshing of superficial cutaneous wrinkles.^[36]^

## 4. Application of PLLA

PLLA is used for tissue augmentation, body plasticity, correction of skin relaxation, and collagen tissue regeneration in the regions such as neck, chest, buttocks, abdomen, upper arms, thighs, knees, and hands due to its long-acting and self-regulating mechanism of tissue proliferation (Table 1).^[2,12,13]^ In the field of facial rejuvenation, PLLA has been used for more than 18 years worldwide. Its indications include tissue expansion, contour plasticity, wrinkle correction, and HIV-associated tissue atrophy.^[8,37]^ Since its application in skin rejuvenation, a number of clinical trials and studies have been carried out to fully evaluate the safety and effectiveness of PLLA globally. Li et al used the new PLLA filler to treat patients with lipoatrophy.^[38]^ By measuring the dermal thickness with ultrasound, they found that the skin thickness increased gradually.^[38]^ A study in France included 40 patients with lipodystrophy who used digital photography software to analyze skin photographs and found that skin thickness increased from 2 to 6 months after PLLA injection.^[39]^ A study of 54 PLLA-filled patients in the United States completed a 12-month follow-up.^[13]^ After 12 months, the skin thickness increased by 54.9% through caliper measuring.^[13]^ According to Moyle et al and Valantin et al, PLLA is mainly used in cutaneous cosmetology and treatment of HIV-associated facial lipoatrophy, which not only achieves good cosmetic results but improves the quality of life.^[40,41]^ In 2019, Bohnert et al found that repeated PLLA treatment can improve contour defects as well as skin quality.^[42]^ According to Baroni et al and Mojallal et al, PLLA has positive effect on skin physiological parameters such as hydration, elasticity, transepidermal water loss, and skin quality assessment such as erythema, pigmentation, pore size, lightness and smoothness, which is mainly due to the proliferation of collagen.^[43,44]^ Previous wide range of cutaneous filling always concentrated in hyaluronic acid or lipo filling. In 1934, Carl Meyer and his assistant John Palmer isolated a newly discovered glycosaminoglycan from ox-eye glass and named the substance “hyaluronic acid” (HA).^[45]^ Hyaluronic acid is a natural linear polysaccharide polymer composed of repeating diglucan units of N-acetyl-D-glucosamine and D-glucuronic acid linked by β (1,4) and β (1,3) glycosidic bonds.^[46]^ The functioning time of HA can reach about 1 year with the improvement of cross-linking technology.^[47]^ However, HA is inactive filler, which means it will require repeated injections no matter how long it lasts for one syringe.^[48]^ Simultaneously, the increasing of injections may lead to high incidence of local cyst formation and local widening figure in regions such as chin, forehead and dorsum.^[49]^ All of the issues mentioned above will affect patients satisfaction. As far as fat injection is concerned, the latest research showed that the survival rate of single injection is 20% -80%.^[50]^ To our understanding, fat may not be an absolute appropriate filler for precise facial volumization nowadays. Excessive injection is needed in order to ensure the survival of local fat, however, which may increase the possibility of embolism.^[51]^ Or large amounts of fat survive locally may resulting in unsatisfactory morphology.^[51]^ Other complications such as necrosis and calcification of fat nodules, granular sensation can also be caused by local excessive injection.^[52]^ Especially for regions such as glabella, forehead, temporal and circumorbital, where cutaneous or subcutaneous injections are applied frequently.^[52]^ HA is easy to induce Tyndall effect locally for it contains cross-linking agent.^[53]^ Meanwhile, deep injection of HA cannot ameliorate superficial cutaneous wrinkles exactly.^[53]^ Some scholars have reported that fat gel may benefited for superficial static wrinkles, but the harvesting process of fat gel is complicated rather than commercialized bring and use filler.^[54–56]^ Besides, fat gel injection also includes the controversies concerning fat injection mentioned above. As we all know, blood vessels densely distribute in regions such as glabella, forehead, temporal and circumorbital, especially in the superficial tissues.^[57]^ At present, no report claimed that accidental intravascular injection can be absolutely avoided in these areas. Therefore, even experienced practitioners still have the risk of embolism concerning HA or fat injection. For PLLA is an injectable material with non-crosslinking agent contained, even if intravascular injection occurs, it will rarely give rise to catastrophic complications such as blindness or large area of skin necrosis.^[58–60]^ Through literature review, we have not yet found any reports relate to embolism caused by PLLA injection in non-HIV patients.^[61]^ Only one case concerning HIV related PLLA embolism be reported in 2012, and the patient already had 10 years of multiple HIV medications intaking.^[61]^ In conclusion, PLLA is a material with extreme safety concerning perspective of embolization. At present, the combination therapy of PLLA and HA is advocated in clinical work.^[62]^ HA and other supportive injection materials can be used for volumization in sub-SMAS spaces and deep fat chambers, while PLLA injection can be used for superficial modification and precise refreshing.^[63]^ Finally, PLLA combined with HA can often achieve the effect of multi-level comprehensive facial rejuvenation.^[63]^

**Table 1:** Applications of PLLA.

Applications of PLLA			
Tissue augmentation	Body plasticity	Correction of skin relaxation	Collagen regeneration

PLLA = Poly-L-lactic acid.

## 5. Complications of PLLA

Through literature reviewing, we found no reports of serious complications associated with PLLA injection in non-HIV patients. According to different reports, the incidences of cutaneous/subcutaneous nodules and granulomas range from 1% to 44%.^[64–68]^ However, with the progress of pharmaceutical technology and injection technology, as well as the improvement of resuspended skills, the probability of nodule formation has been reduced significantly.^[60,65,68]^ Hart et al did not find any cutaneous/subcutaneous nodule in 100 patients receiving thoracic PLLA injections.^[69]^ Other temporary complications, including bruising, swelling, and pain, were significantly relieved within 2 weeks after injection.^[68]^ Reports of PLLA allergy were rare.^[70]^

## 6. Conclusion

PLLA has good effect on improving cutaneous texture, increasing cutaneous thickness and refining static wrinkles et al, which has wide prospect of application in cutaneous rejuvenation of face.

## Author contributions

**Data curation:** Yan Yi.

**Resources:** Yin-Jie Ao.

**Supervision:** Guo-Hui Wu.
